# A computational method for the identification of Dengue, Zika and Chikungunya virus species and genotypes

**DOI:** 10.1371/journal.pntd.0007231

**Published:** 2019-05-08

**Authors:** Vagner Fonseca, Pieter J. K. Libin, Kristof Theys, Nuno R. Faria, Marcio R. T. Nunes, Maria I. Restovic, Murilo Freire, Marta Giovanetti, Lize Cuypers, Ann Nowé, Ana Abecasis, Koen Deforche, Gilberto A. Santiago, Isadora C. de Siqueira, Emmanuel J. San, Kaliane C. B. Machado, Vasco Azevedo, Ana Maria Bispo-de Filippis, Rivaldo Venâncio da Cunha, Oliver G. Pybus, Anne-Mieke Vandamme, Luiz C. J. Alcantara, Tulio de Oliveira

**Affiliations:** 1 Laboratório de Flavivírus, IOC, Fundação Oswaldo Cruz, Rio de Janeiro, Brazil; 2 KwaZulu-Natal Research Innovation and Sequencing Platform (KRISP), College of Health Sciences, University of KwaZuluNatal, Durban, South Africa; 3 Laboratório de Genética Celular e Molecular, ICB, Universidade Federal de Minas Gerais, Belo Horizonte, Minas Gerais, Brazil; 4 Artificial Intelligence Lab, Department of Computer Science, Vrije Universiteit Brussel, Brussels, Belgium; 5 KU Leuven—University of Leuven, Department of Microbiology and Immunology, Rega Institute for Medical Research, Clinical and Epidemiological Virology, Leuven, Belgium; 6 Department of Zoology, University of Oxford, Oxford, United Kingdom; 7 Evandro Chagas Institute, Ministry of Health, Ananindeua, Brazil; 8 Laboratório de Patologia Experimental, Fundação Oswaldo Cruz, Salvador, Brazil; 9 Center for Global Health and Tropical Medicine, Unidade de Microbiologia, Instituto de Higiene e Medicina Tropical, Universidade Nova de Lisboa, Lisbon, Portugal; 10 EMWEB (private company), Herent, Belgium; 11 Division of Vector-Borne Diseases, Centers for Disease Control and Prevention, San Juan, Puerto Rico, United states of America; 12 Coordenação de Vigilância em Saúde e Laboratórios de Referências, Fundação Oswaldo Cruz, Rio de Janeiro, Brazil; University of California San Francisco, UNITED STATES

## Abstract

In recent years, an increasing number of outbreaks of Dengue, Chikungunya and Zika viruses have been reported in Asia and the Americas. Monitoring virus genotype diversity is crucial to understand the emergence and spread of outbreaks, both aspects that are vital to develop effective prevention and treatment strategies. Hence, we developed an efficient method to classify virus sequences with respect to their species and sub-species (i.e. serotype and/or genotype). This tool provides an easy-to-use software implementation of this new method and was validated on a large dataset assessing the classification performance with respect to whole-genome sequences and partial-genome sequences. Available online: http://krisp.org.za/tools.php.

## Author summary

Dengue (DENV), Chikungunya (CHIKV) and Zika (ZIKV) are considered major public health challenges. In addition to the epidemic caused by DENV, which has been described in many tropical countries, the introduction of CHIKV and ZIKV in these countries is a major public health concern. These arboviruses are primarily transmitted by mosquitoes of the species *Ae*. *Aegypti* and its related diseases result in increased financial costs associated with diagnosis and treatment. To support the design of efficient diagnosis, prevention and treatment strategies, a bioinformatics tool has been developed for the genotyping of these viruses based on appropriate evolutionary models in an automatic, accurate and rapid manner. A set of virus reference sequences was obtained from GenBank and used for the development of the tools. This process involved the alignment of the reference sequences followed by phylogenetic tree reconstructions. To assign the genotypes uploaded by the user, the tool analyses the sequences one by one, genotypes through identification, alignment and phylogenetic reconstruction. This computational method allows the high-throughput classification of these virus species and genotypes in seconds. As shown experimentally, genotypes are classified most confidently using the envelope gene or complete genome sequences.

## Introduction

In the recent years, an increasing number of outbreaks of Dengue (DENV), Chikungunya (CHIKV) and Zika (ZIKV) viruses have been reported in Asia and the Americas [1–3]. The predominant mosquito species transmitting DENV, CHIKV and ZIKV, are *Aedes aegypti* and *Aedes Albopictus*, which are widely distributed in tropical and sub-tropical regions [4]. In the past few years, several studies have reported concurrent outbreaks of DENV, CHIKV and ZIKV in the same geographical area [5, 6]. Currently, unprecedented outbreaks of DENV, CHIKV and ZIKV are co-occurring in Brazil. In 2017, the Brazilian Ministry of Health estimated that approximately 251,000 suspected cases of DENV, 185,000 suspected cases of CHIKV and close to 18,000 suspected ZIKV cases had occurred in Brazil [7].

Monitoring virus genotype diversity is crucial to understand the emergence and spread of outbreaks, both aspects that are vital to develop effective prevention and treatment strategies. Both DENV and CHIKV epidemics are associated with a mortality and morbidity that puts a significant economic burden on the affected regions [8,9]. While infections with ZIKV are rarely fatal, as stated before, ZIKV infections may result in Guillain-Barré syndrome and congenital malformations [10,11]. Genomic surveillance of epidemics at the appropriate resolution and consistently classifying the reported genetic sequences, also enables the identification of strains associated with greater epidemic potential [12] or disease severity [13].

However, methods that consistently classify arbovirus sequences at the level of species and sub-species (i.e. serotype and/or genotype) are currently lacking. Additionally, whole genome sequences are often not available in routine clinical settings, forcing the use of shorter gene sequences to classify at viral species or sub-species level. It has however insufficiently been explored which genomic regions are most suitable for accurate classification.

A new computational method for the identification of DENV/CHIKV/ZIKV sequences, with respect to species and sub-species (i.e. serotype and/or genotype), is presented. The classification method is implemented in the Genome Detective software tool, which was validated on a large dataset by assessing the classification performance of whole-genome sequences, partial-genome sequences and products from next-generation sequencing methods. Furthermore, the suitability of different genomic regions for virus classification was evaluated.

## Materials and methods

### Datasets

#### Global whole-genome sequence dataset (Global-WG)

A dataset of previously published whole-genome sequences from GenBank [14] was compiled. This dataset consists out of 4,118 DENV sequences, 653 CHIKV sequences and 413 ZIKV sequences and contains DENV sequences for each of the four known serotypes: DENV-sero1 (n = 1688), DENV-sero2 (n = 1317), DENV-sero3 (n = 897) and DENV-sero4 (n = 216). The list of GenBank accession numbers for this global whole-genome dataset is available in the Supporting Information section (S1 File). In the remainder of this manuscript, this dataset will be referred to as Global-WG.

#### Global envelope sequence dataset (Global-ENV)

A dataset of previously published envelope sequences from GenBank [14] was compiled. This dataset consists out of 4,118 DENV sequences, 2,531 CHIKV sequences and 413 ZIKV sequences and contains DENV sequences for each of the four known serotypes: DENV-sero1 (n = 1688), DENV-sero2 (n = 1317), DENV-sero3 (n = 897) and DENV-sero4 (n = 216). The list of GenBank accession numbers for this global envelope dataset is available in the Supporting Information section (S2 File). In the remainder of this manuscript, this dataset will be referred to as **Global-ENV**.

#### Identification of genotypes and selection of reference sequences

To identify the viral genotypes, a multiple sequence alignment was constructed with the MAFFT alignment software [15] per virus species, using the **Global-WG** dataset. Each alignment was edited manually until a codon-correct alignment was achieved in all genes. The next step in this exploration involved a phylogenetic analysis using PhyML (i.e. Maximum likelihood, 1000 bootstrap replicates) and MrBayes (i.e. Bayesian) [16,17]. With this approach, four main DENV clades (i.e. serotype 1 to 4) and 19 genotypes (i.e. 1I, 1II, 1III, 1IV, 1V, 2I, 2II, 2III, 2IV, 2V, 2VI, 3I, 3II, 3III, 3V, 4I, 4II, 4III and 4IV) were identified. These findings are in agreement with the current consensus in DENV classification [18–21]. For CHIKV, three phylogenetic clades can be distinguished: The East-Central-South African (ECSA) genotype, the Asian-Caribbean genotype and the West African genotype. The West African genotype being more divergent and less widespread than the ECSA genotype and the Asian-Caribbean genotype [22,23]. ZIKV, as well, can be classified into two genotypes. The African genotype, originally identified in Uganda in 1947 [24], is found in many African countries [25]. The Asian genotype was identified in Malaysia in 1966 [26], this genotype has recently caused the worldwide epidemic in Asia and the Pacific [27,28], and is responsible for the epidemic in the Americas [5].

The accuracy and consistency with which a method identifies viral species and genotype clades depends on the selection of a set of representative reference sequences [29–31].

The initial step in the selection of reference strains for our method involved the identification of highly divergent but equidistant whole-genome sequences that are representative for the diversity within the different DENV, CHIKV and ZIKV genotypes, by screening all published complete genome sequences in our **Global-WG** dataset. For example, we normally start by selecting 5–10 sequences that represent the diversity of each virus genotypes. Sequences that met these selection criteria were quality controlled for the presence of insertions, deletions, frame shifts and non-IUPAC characters using VIRULIGN [32]. For DENV, we used the reference sequences that are included with the VIRULIGN software, for ZIKV, we used the reference sequence presented in [33], and for CHIKV we constructed a new reference sequence from NC_004162 that we added to the VIRULIGN repository. Sequences that pass the quality control were aligned using MAFFT [15], and were subjected to phylogenetic analysis using PAUP* (i.e. Neighbor Joining), MrBayes (i.e. Bayesian) and PhyML (i.e. Maximum likelihood) [16,17,34,35] using GTR+G+I. Sequences that gave consistent topologies using all three tree inference methods were retained as potential reference sequences (see Supporting Information, S1 Table) and used in the next step of the evaluation process.

We established that none of the selected reference strains were recombinants (S2 Fig) using the recombination detection program RDP4 [36]

#### Suitability of sub-genomic regions for genotyping purposes

The reference strain dataset (S1 Table) was then explored to establish the suitability of sub-genomic regions for automated genotyping. Two different methods were used.

The first was a boot-scanning method, using a sliding window approach exploring the range between 200 and 2,000 nucleotides. All windows across the genome were used for the construction of Neighbor joining trees with 1,000 bootstrap replicates. The aim was to find the size and segments of the genome that would correctly classify a query sequence with a bootstrap support of >70%.

The second method involved the calculation of the phylogenetic signal present in each of the DENV, CHIKV and ZIKV genes, using the same set of reference sequences. To compute the phylogenetic signal, the TreePuzzle software [37] implementation of the likelihood-mapping method [38] was used. Only between-genotype quartets were evaluated. Quartet puzzling essentially is a three-step procedure, first reconstructing all possible quartet maximum likelihood trees (maximum-likelihood step), then repeatedly combining the quartet trees to an overall tree (puzzling step), and finally computing the majority rule consensus of all intermediate trees giving the quartet puzzling tree (consensus step).

### Classification method and implementation

#### Classification method

Our method involves a viral classification pipeline, drawing inspiration from the one described previously to classify HIV, hepatitis C virus and human T-lymphotropic virus sequences [29,30]. The classification pipeline presented here consists of two classification components. The first classification component enables species and sub-species assignments. The classification analysis subjects a query sequence to a BLAST analysis against a set of reference sequences [39]. A query is assigned to a particular type when BLAST reports an assignment with a score that exceeds a predefined threshold.

The second classification component involves the construction of a Neighbor Joining phylogenetic tree. This component enables assignments on genotype and/or subtype level. First, the query sequence is aligned with a set of reference sequences.

The alignment is produced using the profile alignment option in the ClustalW software [40], such that the query sequence is added to the existing alignment of reference sequences. Subsequent to the alignment, a Neighbor Joining phylogenetic tree, with 100 bootstrap replicates, is constructed. The tree is constructed using the HKY distance metric with gamma among-site rate variation, as implemented in the PAUP* software [34]. The query sequence is assigned to a particular genotype if it clusters monophyletically with that genotype clade with bootstrap support >70%. If the bootstrap support is <70%, the genotype is reported to be unassigned.

#### Software implementation

**Fig 1:**
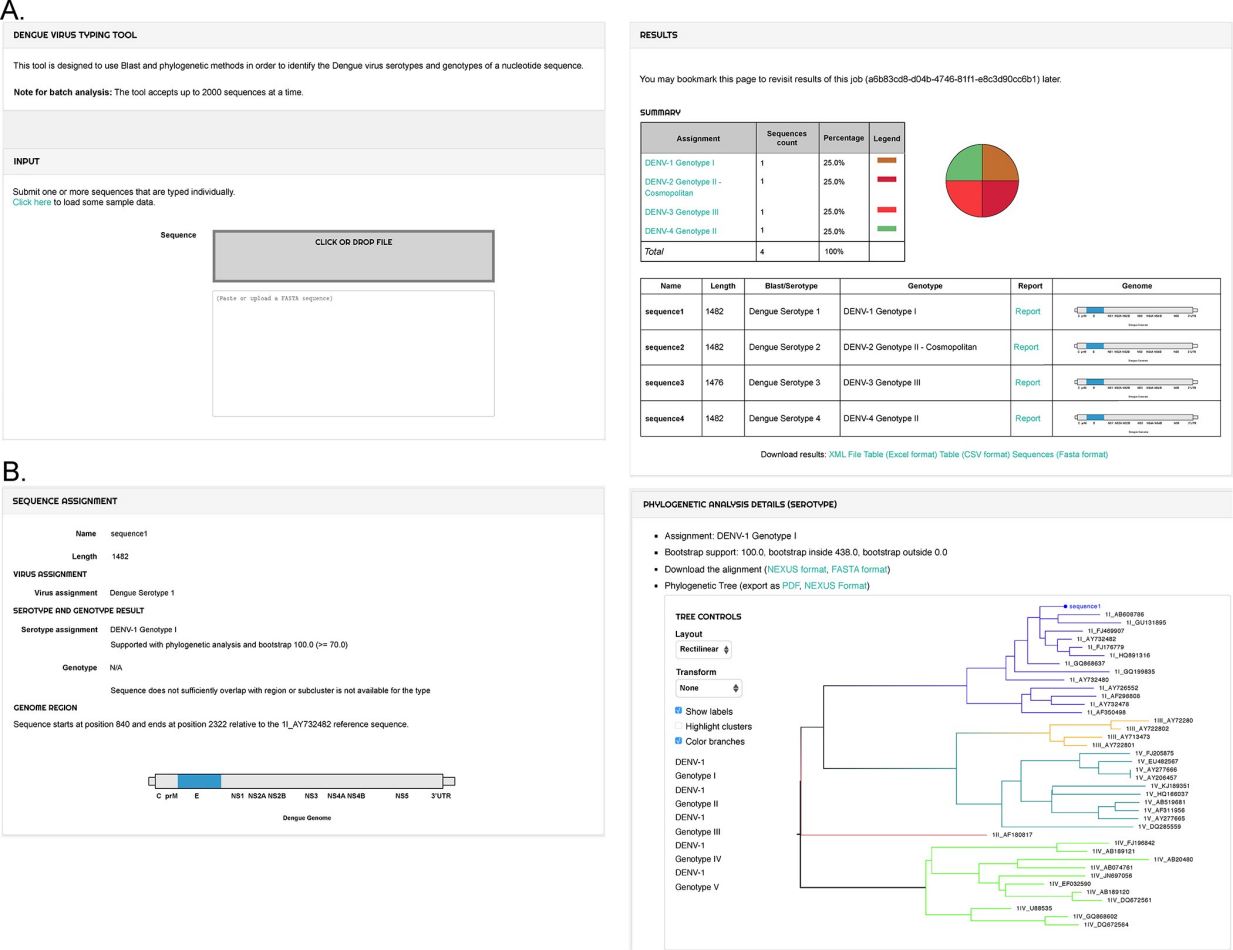
The typing tools’ web interface. The web interface provides users a portal to run classifications on their sequences and to visualize the classification results (A). The typing report presents information about the sequence name of the query sequence, the nucleotide length of the sequence, an illustration of the position of the sequence in the virus’ genome, the species assignment and the genotype assignment. A detailed report is provided for the phylogenetic analysis that resulted into this classification. All results can be exported to a variety of file formats (XML, CSV, Excel or FASTA format). The detailed HTML report (B) contains information on the sequence name, length, assigned virus and genotype, an illustration of the position of the sequence in the virus’ genome and the phylogenetic analysis section. The phylogenetic analysis section shows the alignment and constructed phylogeny: the query sequence is always shown at the top of the phylogenetic tree.

While the classification method was inspired by the one previously presented [29], a new software framework was developed to be easily adaptable to the classification procedures for various viral pathogens. All source code is written in the Java programming language (Fig 1). The software framework is part of the Genome Detective toolchain [41]

#### ArboTyping classification method and implementation

Firstly, the viral species is determined using BLAST, classifying the sequence as DENV, CHIKV or ZIKV.

In case the submitted sequence was assigned either as ZIKV or CHIKV, a Neighbor joining tree is inferred to determine the respective ZIKV or CHIKV genotype. Only for DENV, another BLAST procedure is invoked to assign the serotype first. Based on the inferred serotype, a serotype specific Neighbor joining tree is constructed to determine the Dengue genotype.

**Fig 2:**
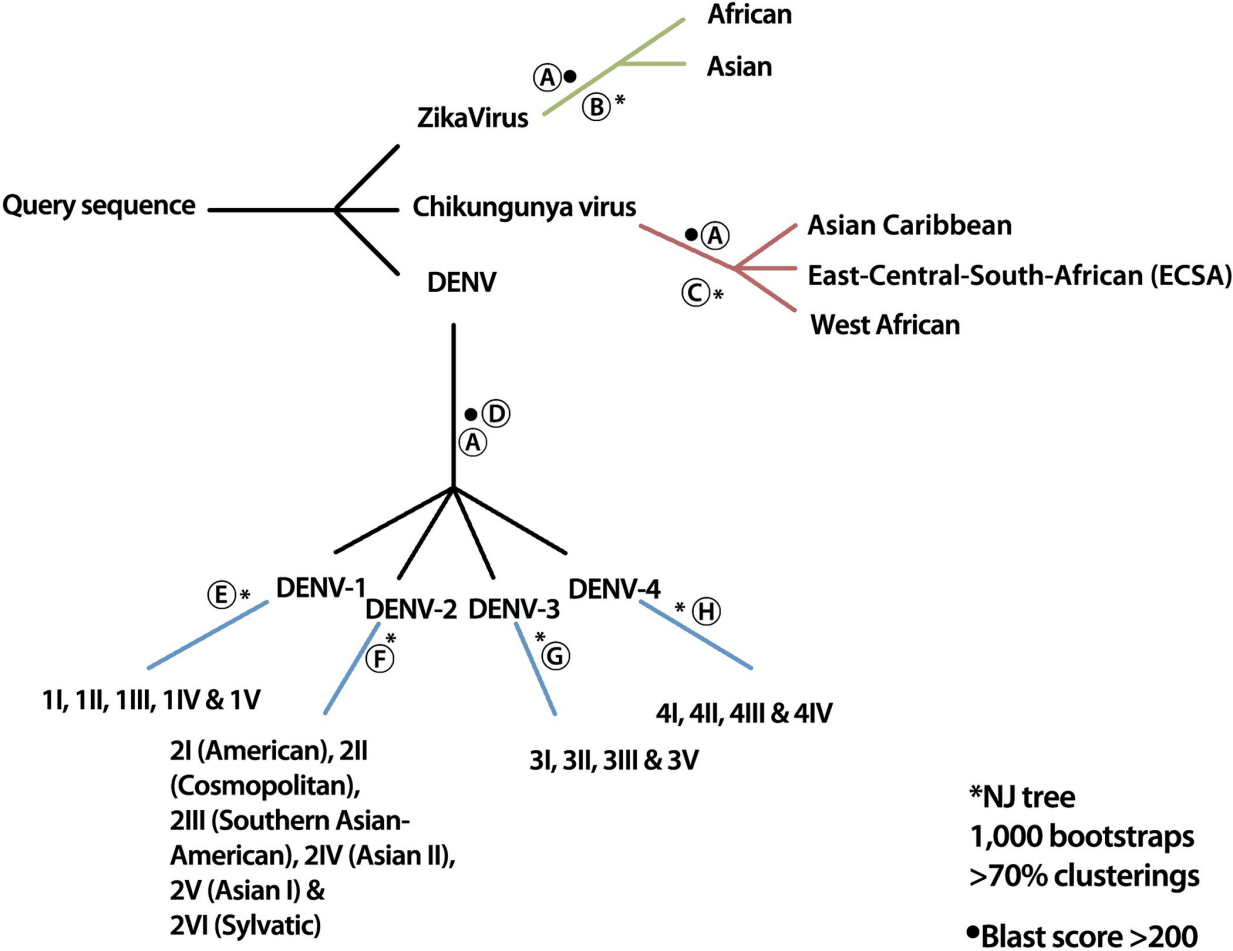
Outline of the classification procedure. Firstly (A), the viral species is determined using BLAST. When the submitted sequence is a *Zika virus*, a Neighbor joining tree is constructed to determine the Zika genotype (B). When the submitted sequence is a *Chikungunya virus*, a Neighbor joining tree is constructed to determine the Chikungunya genotype (C). When the submitted sequence is a *Dengue virus*, the serotype is determined using another BLAST invocation (D). Based on the inferred serotype, a serotype specific Neighbor joining tree is constructed to determine the Dengue genotype (E, F, G, H).

For each of these steps, the earlier discussed reference strains were used, with respect to the appropriate typing level (i.e. virus species, serotype or genotype). This process is summarized in a decision tree in Fig 2.

Testing revealed that a BLAST cut-off value of 200 allowed accurate identification of the virus species and DENV serotypes using sequence segments >150 base pairs.

Note that the species and serotype classification procedure are implemented as separate BLAST steps. This enables the tool to efficiently perform large throughput species classification, such as for the classification of next-generation sequencing reads.

An instance of the ArboTyping web application is publically available on a dedicated server (http://krisp.org.za/tools.php). The web interface on this server accepts up to 2,000 whole-genome or partial genome sequences at a time. The tool can be accessed by the Genome Detective interface or by the selection of individual viruses typing tool (i.e. Zika, Dengue and Chikungunya).

#### Classification performance for whole-genomes and sub-genomic regions

To determine the accuracy of the automated method for whole-genome sequences, the method was evaluated on a whole-genome sequence dataset (i.e. **Global-WG** dataset).

As sequences from sub-genomic regions are more commonly available than whole-genome sequences, the method’s accuracy was also evaluated in this context. For this purpose, the envelope sequences in the **Global-ENV** dataset were used for evaluation.

Each of the sequences considered for evaluation was assigned using both the gold standard and the here described automated method. The gold standard, a manual classification consists of performing an assignment using both Bayesian (i.e. MrBayes, assignment with posterior > 90% [17]) and Maximum likelihood (i.e. PhyML, 1000 bootstrap replicates, assignment with > 70% of replicates [16]) phylogenetic analysis. When the assignments generated by both the Bayesian and Maximum likelihood technique match, the classification is confirmed [31].

The sensitivity, specificity and accuracy of our method was calculated for both species assignment and genotyping. Sensitivity was computed by the formula $\frac{TP}{TP+FN}$, specificity by the formula $\frac{TN}{TN+FP}$ and accuracy by the formula $\frac{TP+TN}{TP+FP+FN+TN}$ [42]. In these formulas: TP = True Positives, FP = False Positives, TN = True Negatives and FN = False Negatives.

## Results

### ArboTyping classification method and implementation

An efficient method to classify virus sequences with respect to their species and sub-species (i.e. serotype and/or genotype) was developed. This method was implemented in Java and this implementation was integrated in an easy-to-use web interface. A detailed description of the method and its implementation can be found in the ‘Classification method and implementation’ Methods subsection.

### Suitability of sub-genomic regions for genotyping purposes

Two different methods were used to verify the suitability of sub-genomic regions for genotyping purposes: a boot-scanning method and a likelihood-mapping method (see Methods).

**Fig 3:**
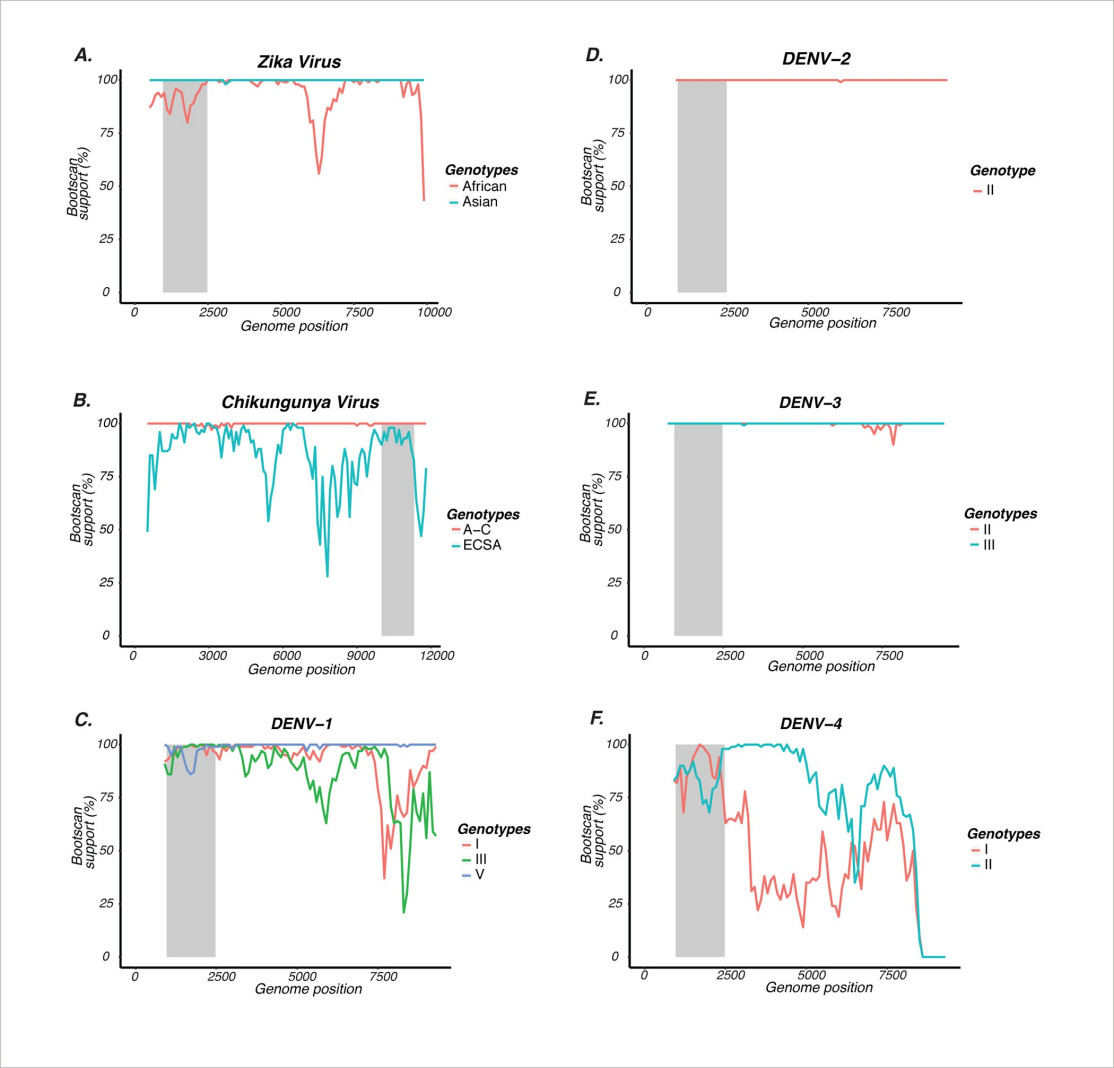
Investigating the suitability of sub-genomic regions for genotyping through boot-scanning. This plot was constructed using bootstrap results from Neighbor Joining trees (1000 bootstrap replicates), performed on the dataset of the indicated reference strains. The boot-scanning method uses a sliding window of a 1500 base pair segment that moves with steps of 100 base pairs along the genome. The X-axis represents the nucleotide position in the genome, and the Y-axis represents bootstrap support in percentages. The light-grey rectangular area marks the location of the envelope gene in each graph. The inset color code shows the genotypes defined in each virus species. For ZIKV (A), this is African and Asian. For CHIKV (B), this is Asian Caribbean (A-C), West African (WA) and East-Central-South African (ECSA). For DENV (C-F), the genotype is visualized by a Roman numeral. Only genotypes which showed less than 100% bootstrap support across the genome are shown.

For DENV, the only sub genomic region that supports confident genotype assignment across the four different serotypes was the envelope gene. For CHIKV, the envelope region E1 was the only region that allowed consistent assignment. The boot-scanning analysis showed that for ZIKV, segments of around 1,200–1,500 base pairs support the genotype assignment with bootstrap > 70% (Fig 3). This was the case over the entire genome, with the exception of the end of the genome (i.e. the non-coding region) and near the NS3 region, where bootstraps fell below 60%.

Our likelihood-mapping analyses show that for DENV, the envelope, NS1, NS3 and NS5 had good phylogenetic signal across all four serotypes. For CHIKV, the envelope E2 gene had the best signal but this region did not provide good boot-scanning support for the classification of the ECSA genotype (Fig 3). For ZIKV, the envelope, NS1, NS2A, NS3, NS4A, NS4B and NS5 regions had good phylogenetic signal. A detailed overview of the results of the likelihood-mapping analysis can be found in the S2 Table of the Supporting Information.

In summary, these analyses show that the envelope genes of the reference datasets of the three pathogens (DENV, 1,485 nucleotides; CHIKV, 1,317 nucleotides; ZIKV, 1,525 nucleotides) are the most suitable targets for reliable genotype classification.

### Classification performance for whole-genome sequences

Our automated method provided specificity, sensitivity and accuracy of 100% for the identification of complete genomes for all viral species and genotypes compared to the gold standard, a manual classification. For a detailed overview of the DENV, CHIKV and ZIKV assignment performance, we refer to the Supporting Information S3 Table.

Only ten of 4118 DENV whole-genomes could not be classified at the genotype level, either by manual phylogenetic analysis or by our automated method. Notably, the seven sequences (AF298807, KF864667, EU179860, JQ922546, KF184975, KF289073, EF457905 of DENV-Sero1 were outliers in the phylogenetic tree (see Supporting Information, S1 Fig). We tested all ten sequences for recombination using boot-scanning (see Supporting Information, S2 Fig) and the recombination detection program RDP4 [36]. We only found sequence AY496879 to be a clear recombinant of DENV genotype 3I and 3II. The two other sequences (DENV-Sero2 KF744408 and DENV-Sero3 JF262783) were also identified as a divergent outlier.

### Classification performance for sub-genomic regions

Our analysis shows that the classification results for the envelope sub-genomic region at the species and genotype level were similar to that obtained using whole-genome sequences and largely in agreement with the gold standard, a manual classification.

For DENV, most of the genotypes were classified with great accuracy (i.e. specificity and sensitivity *>* 99%) using the envelope gene. The exception was DENV-sero2 genotype IV, of which 41 envelope sequences were available and for which 33 were correctly identified (i.e. sensitivity 80.49%, specificity 100%). The CHIKV sequences covering the E1 region were accurately classified for all three genotypes (i.e. 100% sensitivity and specificity). All the ZIKV envelope sequences were classified with 100% sensitivity and specificity. For a detailed overview of the DENV, CHIKV and ZIKV assignment performance refer to Supporting Information S4 Table.

Since a good phylogenetic signal was reported for the DENV and ZIKV NS5 region and the CHIKV E2 region, a classification analysis was performed for these regions as well. For the DENV NS5 region a sensitivity of 57,48% and specificity of 31,35%) was observed. Nearly all ZIKV NS5 sequences were correctly assigned to the African genotype (i.e. sensitivity of 97.72% and specificity of 100%). This indicates that the ZIKV NS5 region might also be used for genotype classification. For CHIKV, the E2 region showed perfect accuracy, similar to the E1 region (i.e. specificity and sensitivity of 100%). However, our previous boot-scanning support showed that the genetically variable E2 region may cause problems for some strains to be correctly identified as ECSA genotype.

In summary, our results suggest that the envelope region of DENV and ZIKV and the E1 envelope region of CHIKV are suitable for genotyping purposes. In addition, these regions contain the largest number of sequences in public databases, which easily allows for a wide range of comparative analyses and validation experiments.

## Discussion

Emerging infectious diseases caused by viral pathogens still represent a major threat to public health worldwide, as recently demonstrated by outbreaks of Ebola, Zika, Middle East Respiratory Syndrome (MERS) and Yellow Fever virus. Fast and accurate real-time monitoring of outbreaks and surveillance of on-going epidemics is crucial to anticipate viral spread and to design effective prevention or treatment strategies. To this end, an accurate and reliable method for the classification of ZIKV/DENV/CHIKV arboviruses was developed: The ArboTyping tool.

The ArboTyping tool implements a classification pipeline that consists of a BLAST-based species assignment and phylogenetic assessment to identify subspecies (i.e. genotypes) with respect to a set of reference strains, as exemplified for other virus species by previous work [29–31]. To enable accurate classification, a set of reference sequences that cover the extent of diversity within species and subspecies, was carefully selected.

The classification performance of the ArboTyping tool was assessed on a dataset of whole-genome sequences. All whole-genome sequences in this dataset that could be confidently assigned a species and genotype with the gold standard, a manual classification procedure, were concordant with the typing tool.

There were, however, 10 sequences that could not be classified using the manual classification procedure: further analyses show that these 10 sequences consist out of 3 outlier sequences, 2 clades of outlier sequences (3 sequences in each outlier clade) and 1 recombinant sequence. As these outliers have been previously identified [43], these results need to be further investigated to assess whether these outliers form new genotypes [44].

However, whole-genome sequences are currently not routinely available and the suitability of the different genomic regions was evaluated with respect to their use for classification. Since the envelope gene is a popular target for phylogenetic classification, there is a large availability of envelope sequences in public databases. Therefore, the performance of the ArboTyping tool was evaluated on a large dataset of envelope sequences (i.e. **Global-ENV** dataset). For these envelope sequences, a classification performance close to the tool’s performance on whole-genome sequences was reported.

While the availability of sequence products originating from other genomic regions is currently low, it can be expected that these regions will increase in relevance given the interest in developing antiviral agents that target non-structural proteins. Therefore, more detailed studies to assess the classification performance of other genomic regions are warranted [44].

In this manuscript, we focus on the classification of consensus sequences on the species and sub-species level. However, Genome Detective, the framework in which our tools are integrated, is also a virus discovery toolchain [41]. Genome Detective’s user interface allows users to supply raw next-generation sequence reads that can be automatically assembled into a consensus and passed to the ArboTyping tool. Details on the methods used to assemble reads in Genome Detective and an extensive validation using raw NGS reads can be found in [41].

In conclusion, the new method presented here allows the fast, accurate and high-throughput classification of DENV, CHIKV and ZIKV species and genotypes. Species can be classified using different sequencing products (i.e. whole-genome sequences, envelope sequences and individual next-generation sequencing reads) and genotypes can be classified most confidently when using envelope sequences or whole-genome sequences. This method accommodates the need to consistently and accurately classify DENV/CHIKV/ZIKV sequences, which is essential to implement epidemic tracing and to support outbreak surveillance efforts. Additionally, we present a solid framework that has the potential to serve as the foundation for many other arbovirus classification tools. These tools are also useful to be integrated in data management environments [45].

Our method is implemented in the Genome Detective software framework, suitable for many virus typing tools. The web application that makes our tool available through an easy-to-use web interface is available online via a dedicated server that is hosted at http://www.krisp.org.za/tools.php.

## Supporting information

S1 Fig Maximum likelihood phylogenetic tree of the DENV-sero1 outliers. All full genome DENV-sero1 sequences were assigned to genotype-level using manual phylogenetic analysis and classification by the automated typing tool. In total, seven full genomes of DENV-sero1 could not be classified at genotype level by either classification method. These seven sequences are visualized in a phylogenetic tree of the WGS datasets, colored according to genotype. (1I in blue, 1II in green, 1III in red, 1IV in yellow, 1V in pink) It can be seen that a divergent cluster of six genomes (AF298807, KF864667, KF184975, EU179860, KF289073 and JQ922546 in black) form an outlier clade and one genome (EF457905 in black) can be considered an outlier. However, note that these seven genomes could be properly assigned to serotype 1.
(TIF)

S2 Fig Recombination analysis for the DENV whole genome sequences. The bootscan results for the ten whole genomes of DENV that could not be classified at genotype level are shown. Boot-scanning analysis was performed using a window length of 1500 base pairs and a step size of 100 base pairs. The different colours represent the genotypes for each serotype. The X-axis represents the nucleotide position in the genome and the Y-axis represents bootstrap results in percentages. In total, 7 DENV-sero1 sequences were analysed and 1 sequence for each of the other serotypes, i.e. DENV-sero2, DENV-sero3 and DENV-sero4. We only found sequence AY496879 to be a recombinant of DENV genotype 3I and 3II. The other sequences are outliers (i.e. JF262783, KF744408, EF457905) or clades of outliers (i.e.: AF298807, KF864667 and KF184975 form an outlier clade; EU179860, KF289073 and JQ922546 form an outlier clade).
(TIF)

S1 Table Reference strains selected for the DENV, CHIKV, ZIKV genotypes. These reference sequences were selected to be representative for the diversity within the different DENV, CHIKV and ZIKV genotypes that circulate within these virus species.
(DOCX)

S2 Table Phylogenetic signal estimated by likelihood mapping for DENV (DENV-sero1 to DENV-sero4), CHIKV and ZIKV sub-genomic regions. Phylogenetic signal was calculated separately per protein by the likelihood mapping method implemented in the software TreePuzzle. Likelihood mapping analysis computes the likelihood of the three possible trees that can be constructed from all possible inter-genotype quartets of taxa. The results for the resolved quartets and unresolved quartets are shown in the table, while the partially resolved quartets are not listed (can be obtained by 100%—(un)resolved quartets). Partially resolved quartets represent the quartets for which conflicting phylogenetic signal or potential recombination is present. Genomic regions for which the percentage of resolved quartets is higher than 90% are shaded in orange and are considered to be characterized by sufficient phylogenetic signal.
(DOCX)

S3 Table Evaluation of the automated phylogenetic method to classify DENV, CHIKV and ZIKV whole-genome genomes. The new classification method consists of 2 parts: determining the species (and for DENV also the serotype) using a BLAST procedure, followed by determining the genotype using an automated phylogenetic method. Our method was able to assign all sequences in the whole-genome validation dataset to the right species and DENV serotype. Therefore, in this table, we focus on the classification performance with respect to genotype assignment, based on the output of the BLAST step (i.e. a dataset of the proper species and serotype). The classification results were compared to manual phylogenetic analysis. Column names: TP = total positives, TN = total negatives, FP = false positive, FN = false negative, SENS = sensitivity, SPEC = specificity, ACC = accuracy.
(DOCX)

S4 Table Evaluation of the automated phylogenetic method to classify DENV, CHIKV and ZIKV envelope genomes. The new classification method consists of 2 parts: determining the species (and for DENV also the serotype) using a BLAST procedure, followed by determining the genotype using an automated phylogenetic method. Our method was able to assign all sequences in the envelope validation dataset to the right species and DENV serotype. Therefore, in this table, we focus on the classification performance with respect to genotype assignment, based on the output of the BLAST step (i.e. a dataset of the proper species and serotype). The classification results were compared to manual phylogenetic analysis. Column names: TP = total positives, TN = total negatives, FP = false positive, FN = false negative, SENS = sensitivity, SPEC = specificity, ACC = accuracy.
(DOCX)

S1 File Accession number of the sequences collected from DENV, ZIKV and CHIKV whole-genome genomes. A GenBank mining of sequences was performed against whole-genome genomes of these viruses that had the genotype reported for sensitivity, specificity and accuracy tests of the tool.
(XLSX)

S2 File Accession number of the sequences collected from DENV, ZIKV and CHIKV envelope genomes. A GenBank mining of sequences was performed against envelope genomes of these viruses that had the genotype reported for sensitivity, specificity and accuracy tests of the tool.
(XLSX)

## References

[1] Bhatt S, Gething PW, Brady OJ, Messina JP, Farlow AW, Moyes CL, et al The global distribution and burden of dengue. Nature. 2013;496:504–507. 10.1038/nature12060 23563266 PMC3651993

[2] Weaver SC, Lecuit M. Chikungunya Virus and the Global Spread of a Mosquito-Borne Disease. New England Journal of Medicine. 2015;372(13):1231–1239. 10.1056/NEJMra1406035 25806915

[3] Fauci AS, Morens DM. Zika Virus in the Americas—Yet Another Arbovirus Threat. New England Journal of Medicine. 2016;374(7):601–604. 10.1056/NEJMp1600297 26761185

[4] Kraemer MUG, Sinka ME, Duda KA, Mylne AQN, Shearer FM, Barker CM, et al The global distribution of the arbovirus vectors Aedes aegypti and Ae. Albopictus. eLife. 2015;4(08347). 10.7554/eLife.08347 26126267 PMC4493616

[5] Cardoso CW, Paploski IAD, Kikuti M, Rodrigues MS, Silva MMO, Campos GS, et al Outbreak of Exanthematous Illness associated with Zika, Chikungunya, and Dengue viruses, Salvador, Brazil. Emerging Infectious Diseases. 2015;21(12):2274–2276. 10.3201/eid2112.151167 26584464 PMC4672408

[6] Roth A, Mercier A, Lepers C, Hoy D, Duituturaga S, Benyon E, et al Concurrent outbreaks of dengue, chikungunya and Zika virus infections-an unprecedented epidemic wave of mosquito-borne viruses in the Pacific 2012–2014. Euro Surveill. 2014;19(41):20929 10.2807/1560-7917.ES2014.19.41.20929. 25345518

[7] Ministério de Saúde B. Boletim Epidemiológico Secretaria de Vigilância em saúde; 2018 v.49.

[8] Shepard DS, Undurraga EA, Halasa YA, Stanaway JD. The global economic burden of dengue: A systematic analysis. Lancet Infect Dis. 2016;16(8):935–941. 10.1016/S1473-3099(16)00146-8 27091092

[9] Morens DM, Fauci AS. Meeting the Challenge of Epidemic Chikungunya. Journal of Infectious Diseases. 2016;214(suppl 5):S434–S435. 10.1093/infdis/jiw291 27920168 PMC5137243

[10] Rasmussen SA, Jamieson DJ, Honein MA, Petersen LR. Zika Virus and Birth Defects–Reviewing the Evidence for Causality. New England Journal of Medicine. 2016; p.1–7. 10.1056/NEJMsr1604338 27074377

[11] Brasil P, Sequeira PC, Freitas AD, Zogbi HE, Calvet GA, de Souza RV, et al Guillain-Barrè syndrome associated with Zika virus infection. The Lancet. 2016; 1482 10.1016/S0140-6736(16)30058–7. 27115821

[12] Manokaran G, Finol E, Wang C, Gunaratne J, Bahl J, Ong EZ, et al Dengue subgenomic RNA binds TRIM25 to inhibit interferon expression for epidemiological fitness. Science. 2015;350(6257):217–221. 10.1126/science.aab3369 26138103 PMC4824004

[13] Katzelnick LC, Fonville JM, Gromowski GD, Bustos Arriaga J, Green A, James SL, et al Dengue viruses cluster antigenically but not as discrete serotypes. Science (New York, NY). 2015;349(6254):1338–43. 10.1126/science.aac5017 26383952 PMC4876809

[14] Benson DA, Cavanaugh M, Clark K, Karsch-Mizrachi I, Lipman DJ, Ostell J, et al GenBank. Nucleic acids research. 2013;41(D1):D36–D42. 10.1093/nar/gkr1202 27899564 PMC5210553

[15] Katoh K, Standley DM. MAFFT multiple sequence alignment software version 7: Improvements in performance and usability. Molecular Biology and Evolution. 2013;30(4):772–780. 10.1093/molbev/mst010 23329690 PMC3603318

[16] Guindon S, Gascuel O. A simple, fast, and accurate algorithm to estimate large phylogenies by maximum likelihood. Systematic Biology. 2003;52(5):696–704. 10.1080/10635150390235520 14530136

[17] Ronquist F, Huelsenbeck JP. MrBayes 3: Bayesian phylogenetic inference under mixed models. Bioinformatics. 2003;19(12):1572–1574. 10.1093/bioinformatics/btg180 12912839

[18] Rico-Hesse R. Molecular evolution and distribution of dengue viruses type 1 and 2 in nature. Virology. 1990;174(2):479–493. 10.1016/0042-6822(90)90102-W 2129562

[19] Twiddy SS, Farrar JJ, Vinh Chau N, Wills B, Gould Ea, Gritsun T, et al Phylogenetic relationships and differential selection pressures among genotypes of dengue-2 virus. Virology. 2002;298(1):63–72. 10.1006/viro.2002.1447 12093174

[20] Chungue E, Deubel V, Cassar O, Laille M, Martin PMV. Molecular epidemiology of dengue 3 viruses and genetic relatedness among dengue 3 strains isolated from patients with mild or severe form of dengue fever in French Polynesia. Journal of general virology. 1993;74(12):2765–2770. 10.1099/0022-1317-74-12-2765 8277284

[21] Klungthong C, Zhang C, Mammen MP, Ubol S, Holmes EC. The molecular epidemiology of dengue virus serotype 4 in Bangkok, Thailand. Virology. 2004;329(1):168–179. 10.1016/j.virol.2004.08.003 15476884

[22] Nunes MRT, Faria NR, de Vasconcelos JM, Golding N, Kraemer MU, de Oliveira LF, et al Emergence and potential for spread of Chikungunya virus in Brazil. BMC Medicine. 2015;13(1):102 10.1186/s12916-015-0348-x 25976325 PMC4433093

[23] Volk SM, Chen R, Tsetsarkin KA, Adams AP, Garcia TI, Sall AA, et al Genome-scale phylogenetic analyses of chikungunya virus reveal independent emergences of recent epidemics and various evolutionary rates. Journal of virology. 2010;84(13):6497–6504. 10.1128/JVI.01603-09 20410280 PMC2903258

[24] Dick GWA, Kitchen SF, Haddow AJ. Zika virus (I). Isolations and serological specificity. Transactions of the Royal Society of Tropical Medicine and Hygiene. 1952;46(5):509–520. 10.1016/0035-9203(52)90042-4 12995440

[25] Kindhauser MK, Allen T, Frank V, Santhana RS, Dye C. Zika: the origin and spread of a mosquito-borne virus. Bull World Health Organ. 2016;171082. 10.2471/BLT.16.171082 PMC5034643 27708473

[26] Marchette NJ, Garcia R, Rudnick A. Isolation of Zika virus from Aedes aegypti mosquitoes in Malaysia. American Journal of Tropical Medicine and Hygiene. 1969;18(3):411–415. 10.1056/NEJMp1002530 4976739

[27] Cao-Lormeau VM, Roche C, Teissier A, Robin E, Berry AL, Mallet HP, et al Zika Virus, French Polynesia, South Pacific, 2013. Emerging Infectious Diseases. 2014;20(6):1085–1086. 10.3201/eid2006.140138 24856001 PMC4036769

[28] Hayes EB, Others. Zika virus outside Africa. Emerg Infect Dis. 2009;15(9):1347–1350. 10.3201/eid1509.090442 19788800 PMC2819875

[29] Alcantara LCJ, Cassol S, Libin P, Deforche K, Pybus OG, Van Ranst M, et al A standardized framework for accurate, high-throughput genotyping of recombinant and non-recombinant viral sequences. Nucleic Acids Research. 2009;37(Suppl 2):634–642. 10.1093/nar/gkp455 19483099 PMC2703899

[30] de Oliveira T, Deforche K, Cassol S, Salminen M, Paraskevis D, Seebregts C, et al An automated genotyping system for analysis of HIV-1 and other microbial sequences. Bioinformatics. 2005;21(19):3797–3800. 10.1093/bioinformatics/bti607 16076886

[31] Pineda-Peña AC, Faria NR, Imbrechts S, Libin P, Abecasis AB, Deforche K, et al Automated subtyping of HIV-1 genetic sequences for clinical and surveillance purposes: Performance evaluation of the new REGA version 3 and seven other tools. Infection, Genetics and Evolution. 2013;19:337–348. 10.1016/j.meegid.2013.04.032 23660484

[32] Libin P., Deforche K., Abecasis A. B., & Theys K. (2018). VIRULIGN: fast codon-correct alignment and annotation of viral genomes. *Bioinformatics (Oxford*, *England)*. 10.1093/bioinformatics/bty851 PMC6513156 30295730

[33] Theys K., Libin P., Dallmeier K., Pineda-Peña A. C., Vandamme A. M., Cuypers L., & Abecasis A. B. (2017). Zika genomics urgently need standardized and curated reference sequences. *PLoS pathogens*, 13(9), e1006528 10.1371/journal.ppat.1006528 28880955 PMC5589256

[34] Salemi M, Vandamme AM. The phylogenetic handbook: a practical approach to DNA and protein phylogeny. Cambridge University Press; 2003.

[35] Nylander JAA, Wilgenbusch JC, Warren DL, Swofford DL. AWTY (are we there yet?): a system for graphical exploration of MCMC convergence in Bayesian phylogenetics. Bioinformatics. 2008;24(4):581–583. 10.1093/bioinformatics/btm388 17766271

[36] Martin DP, Lemey P, Lott M, Moulton V, Posada D, Lefeuvre P. RDP3: A flexible and fast computer program for analyzing recombination. Bioinformatics. 2010;26(19):2462–2463. 10.1093/bioinformatics/btq467 20798170 PMC2944210

[37] Ha Schmidt, Strimmer K, Vingron M, von Haeseler A. TREE-PUZZLE: maximum likelihood phylogenetic analysis using quartets and parallel computing. Bioinformatics (Oxford, England). 2002;18(3):502–504. 10.1093/bioinformatics/18.3.502 11934758

[38] Strimmer K, von Haeseler A. Likelihood-mapping: a simple method to visualize phylogenetic content of a sequence alignment. Proceedings of the National Academy of Sciences. 1997;94(13):6815–6819. 10.1073/pnas.94.13.6815 9192648 PMC21241

[39] Altschul SF, Gish W, Miller W, Myers EW, Lipman DJ. Basic Local Alignment Search Tool; 1990. 10.1016/S0022-2836(05)80360-2 2231712

[40] Larkin MA, Blackshields G, Brown NP, Chenna R, Mcgettigan PA, McWilliam H, et al Clustal W and Clustal X version 2.0. Bioinformatics. 2007;23(21):2947–2948. 10.1093/bioinformatics/btm404 17846036

[41] Vilsker M, Moosa Y, Nooij S, Fonseca V, Ghysens Y, Dumon K, Pauwels R, Alcantara LC, Vanden Eynden E, Vandamme AM, Deforche K, de Oliveira T, Bioinformatics (2019), 10.1093/bioinformatics/bty695 30124794 PMC6524403

[42] Banoo S, Bell D, Bossuyt P, Herring A, Mabey D, Poole F, et al Evaluation of diagnostic tests for infectious diseases: General principles. Nature Reviews Microbiology. 2006;4(9 SUPPL.) S21–S31. 10.1038/nrmicro1523 17034069

[43] Libin P., Vanden Eynden E., Incardona F., Nowé A., Bezenchek A.; EucoHIV Study Group, Sönnerborg A., Vandamme A.-M., Theys K., Baele G.(2017). PhyloGeoTool: interactively exploring large phylogenies in an epidemiological context. Bioinformatics, 33(24):3993–3995. 10.1093/bioinformatics/btx535 28961923 PMC5860094

[44] Cuypers L., Libin P.J.K., Simmonds P., Nowé A., Muñoz-Jordán. J., Alcantara L.C.J., Vandamme A.-M., Santiago G.A., Theys K. (2018). Time to Harmonize Dengue Nomenclature and Classification. Viruses, 10(10), pii: E569 10.3390/v10100569 30340326 PMC6213058

[45] Libin P., Beheydt G., Deforche K., Imbrechts S., Ferreira F., Van Laethem K., Theys K., Carvalho A.P., Cavaco-Silva J., Lapadula G., Torti C., Assel M., Wesner S., Snoeck J., Ruelle J., De Bel A., Lacor P., De Munter P., Van Wijngaerden, E., Zazzi M., Kaiser R., Ayouba A., Peeters M., de Oliveira T., Alcantara L.C., Grossman Z., Sloot P., Otelea D., Paraschiv S., Boucher C., Camacho R.J., Vandamme A.-M. (2013). RegaDB: community-driven data management and analysis for infectious diseases. Bioinformatics. 2013, 29(11):1477–80. 10.1093/bioinformatics/btt162 23645815 PMC3661054

