# Supplementary figures and images for: A computational method for the identification of Dengue, Zika and Chikungunya virus species and genotypes
Source: PLoS Negl Trop Dis. 2019 May 8;13(5):e0007231. doi: 10.1371/journal.pntd.0007231 (PMC6527240; doi:10.1371/journal.pntd.0007231)

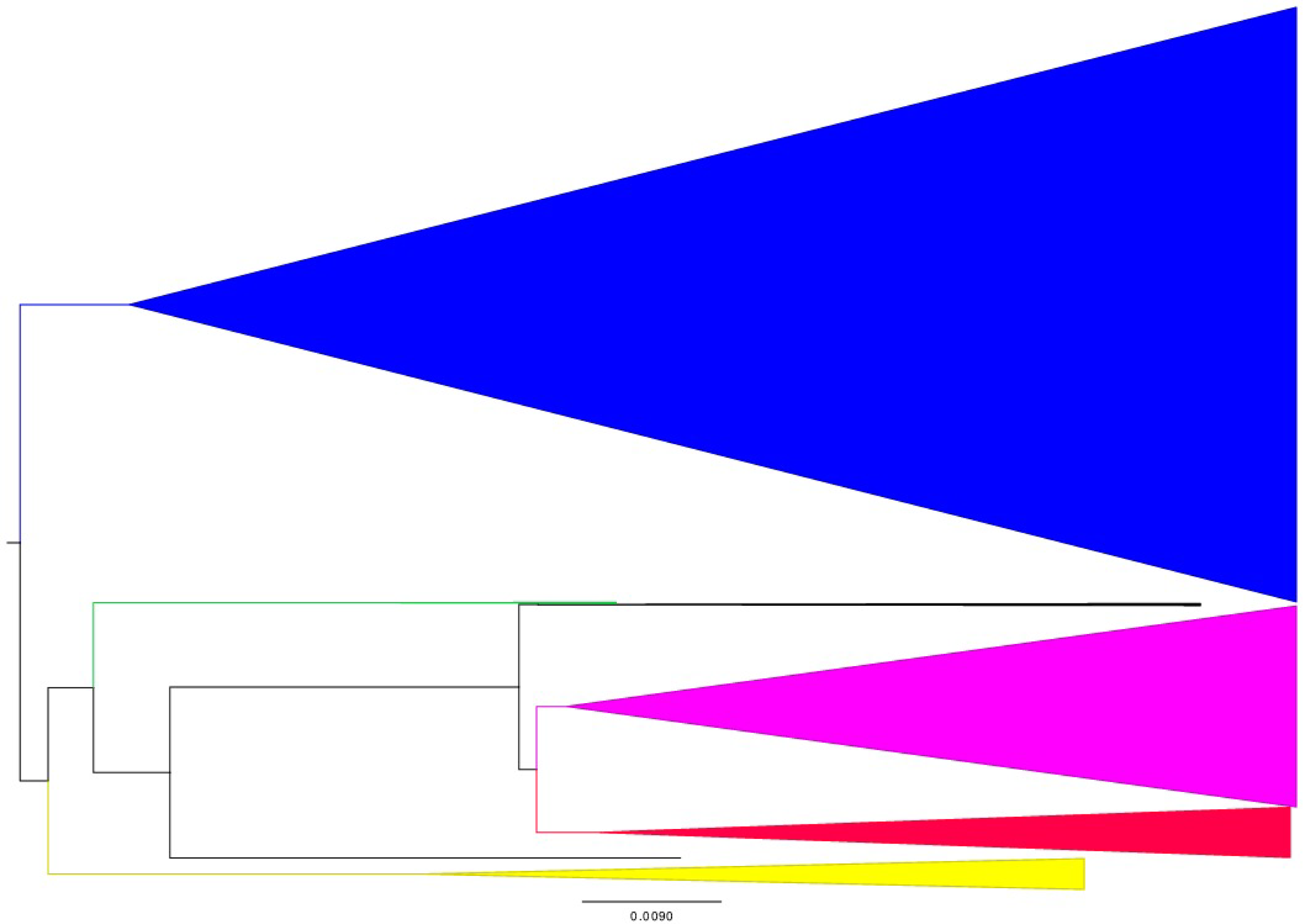

Supplement: S1 Fig — All full genome DENV-sero1 sequences were assigned to genotype-level using manual phylogenetic analysis and classification by the automated typing tool. In total, seven full genomes of DENV-sero1 could not be classified at genotype level by either classification method. These seven sequences are visualized in a phylogenetic tree of the WGS datasets, colored according to genotype. (1I in blue, 1II in green, 1III in red, 1IV in yellow, 1V in pink) It can be seen that a divergent cluster of six genomes (AF298807, KF864667, KF184975, EU179860, KF289073 and JQ922546 in black) form an outlier clade and one genome (EF457905 in black) can be considered an outlier. However, note that these seven genomes could be properly assigned to serotype 1. (TIF) [file pntd.0007231.s001.tif]

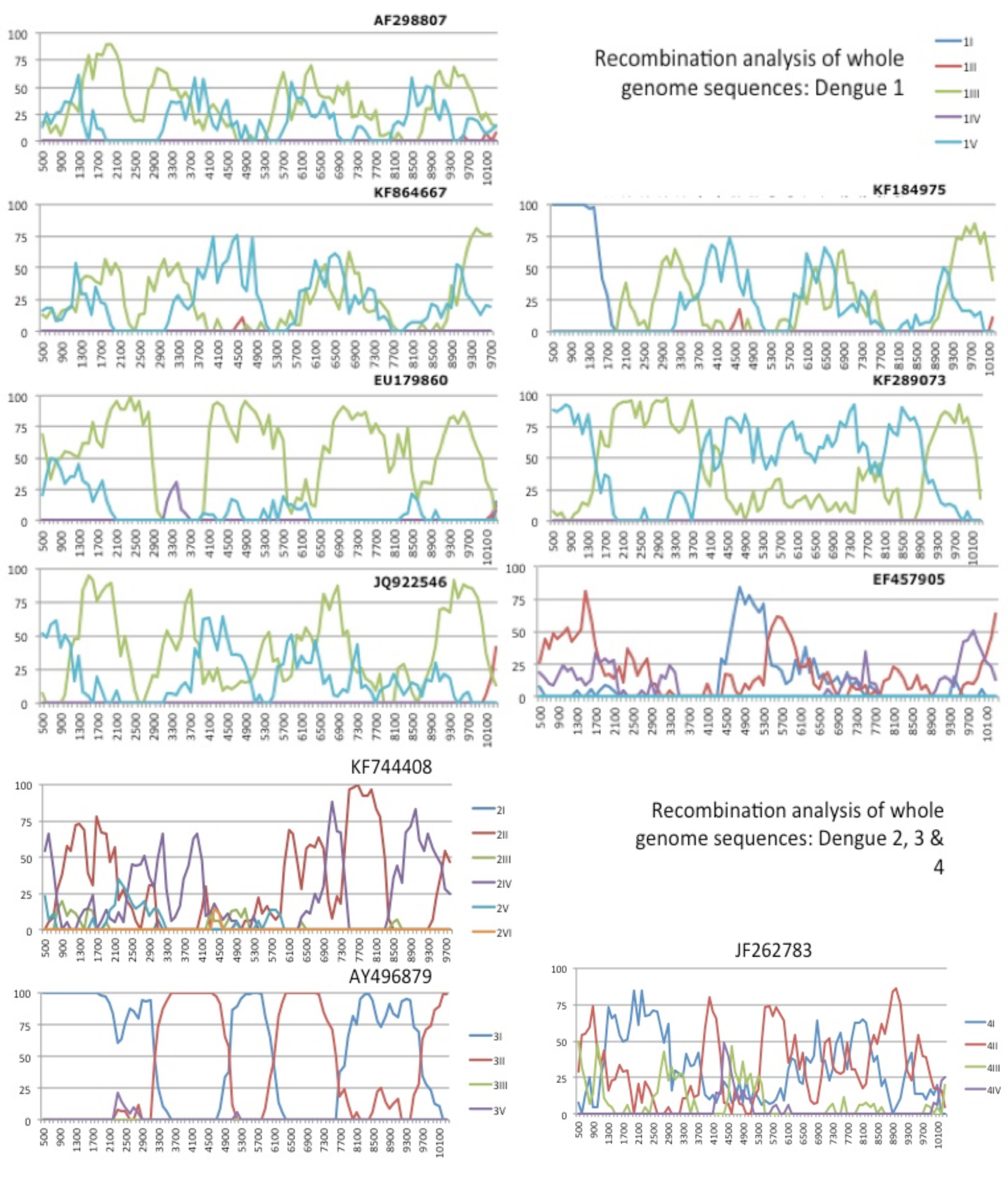

Supplement: S2 Fig — The bootscan results for the ten whole genomes of DENV that could not be classified at genotype level are shown. Boot-scanning analysis was performed using a window length of 1500 base pairs and a step size of 100 base pairs. The different colours represent the genotypes for each serotype. The X-axis represents the nucleotide position in the genome and the Y-axis represents bootstrap results in percentages. In total, 7 DENV-sero1 sequences were analysed and 1 sequence for each of the other serotypes, i.e. DENV-sero2, DENV-sero3 and DENV-sero4. We only found sequence AY496879 to be a recombinant of DENV genotype 3I and 3II. The other sequences are outliers (i.e. JF262783, KF744408, EF457905) or clades of outliers (i.e.: AF298807, KF864667 and KF184975 form an outlier clade; EU179860, KF289073 and JQ922546 form an outlier clade). (TIF) [file pntd.0007231.s002.tif]
