# Supplementary material for: A computational method for the identification of Dengue, Zika and Chikungunya virus species and genotypes
Source: PLoS Negl Trop Dis. 2019 May 8;13(5):e0007231. doi: 10.1371/journal.pntd.0007231 (PMC6527240; doi:10.1371/journal.pntd.0007231)
Supplement: S1 Table — These reference sequences were selected to be representative for the diversity within the different DENV, CHIKV and ZIKV genotypes that circulate within these virus species. (DOCX) [file pntd.0007231.s003.docx]

**S1 Table:** Reference strains selected for the DENV, ZIKV, CHIKV genotypes.

| ZIKV Genotype | Accession Number | Country | Year |
| --- | --- | --- | --- |
| African | AY632535 | Uganda | 1947 |
| African | KF383116 | Senegal | 1968 |
| African | HQ234500 | Nigeria | 1968 |
| African | KF383115 | Cent Afr Rep | 1968 |
| African | HQ234501 | Senegal | 1984 |
| African | KF383117 | Senegal | 1997 |
| African | KF383118 | Senegal | 2001 |
| African | KF383119 | Senegal | 2001 |
| African | KF383121 | Senegal | -N/A- |
| African | KF268950 | Cent Afr Rep | -N/A- |
| African | KF268949 | Cent Afr Rep | -N/A- |
| Asian | HQ234499 | Malaysia | 1966 |
| Asian | EU545988 | Micronesia | 2007 |
| Asian | JN860885 | Cambodia | 2010 |
| Asian | KF993678 | Canada | 2013 |
| Asian | KJ776791 | FrenchPolynesia | 2013 |

| CHIKV Genotype | Accession Number | Country | Year |
| --- | --- | --- | --- |
| Asian | HM045813 | India | 1963 |
| Asian | EF027140 | India | 1963 |
| Asian | EF027141 | India | 1973 |
| Asian | HM045790 | Philipiness | 1985 |
| Asian | FN295483 | Malaysia | 2006 |
| Asian | FJ807897 | Taiwan | 2007 |
| ESCA_IOC | HM045811 | Tanzania | 1953 |
| ESCA_IOC | HM045821 | Senegal | 1963 |
| ESCA_IOC | AM258993 | Reunion | 2005 |
| ESCA_IOC | AM258991 | Seyche | 2005 |
| ECSA_IN | AB455494 | Japan | 2006 |
| ESCA_IOC | EF012359 | Mauri | 2006 |
| ECSA_IN | EU244823 | Italy | 2007 |
| ECSA_IN | FJ445426 | SriLanka | 2008 |
| ECSA_IN | FN295485 | Malaysia | 2008 |
| ECSA_IN | GU199352 | China | 2008 |
| ECSA_IN | GU301781 | Thailand | 2009 |
| ESCA_IOC | HM045784 | Central | -N/A- |
| ESCA_IOC | HM045822 | Central | -N/A- |
| ESCA_IOC | HM045792 | South | -N/A- |
| WestAfr | HM045786 | Nigeria | 1964 |
| WestAfr | HM045785 | Senegal | 1966 |
| WestAfr | HM045815 | Senegal | 1979 |
| WestAfr | HM045817 | Senegal | 2005 |
| WestAfr | HM045818 | Ivory | -N/A- |
| WestAfr | HM045820 | Ivory | -N/A- |

| Dengue Virus 1 Genotype | Accession Number | Country | Year |
| --- | --- | --- | --- |
| 1I | AF350498 | -N/A- | 1980 |
| 1I | AY732478 | Thailand | 1991 |
| 1I | AY732480 | Thailand | 1994 |
| 1I | GQ868637 | Cambodia | 2000 |
| 1I | AY732482 | Thailand | 2001 |
| 1I | FJ469907 | Singapore | 2003 |
| 1I | GQ199835 | VietNam | 2005 |
| 1I | FJ176779 | China | 2006 |
| 1I | AB608786 | Taiwan | 2008 |
| 1I | GU131895 | Cambodia | 2009 |
| 1I | HQ891316 | Sri Lanka | 2009 |
| 1I | AY726552 | Myanmar | 2012 |
| 1I | AF298808 | Djibouti | -N/A- |
| 1II | AF180817 | -N/A- | -N/A- |
| 1III | AY713473 | Myanmar | 1971 |
| 1III | AY722801 | Myanmar | 1976 |
| 1III | AY722802 | Myanmar | 1996 |
| 1III | AY722803 | Myanmar | 1998 |
| 1IV | EF032590 | -N/A- | 1995 |
| 1IV | AB189121 | Indonesia | 1998 |
| 1IV | DQ672564 | USA | 2001 |
| 1IV | DQ672561 | USA | 2001 |
| 1IV | FJ196842 | China | 2003 |
| 1IV | GQ868602 | Philippines | 2004 |
| 1IV | AB204803 | Japan | 2004 |
| 1IV | JN697056 | Malaysia | 2005 |
| 1IV | U88535 | -N/A- | -N/A- |
| 1IV | AB074761 | -N/A- | -N/A- |
| 1IV | AB189120 | Indonesia | -N/A- |
| 1V | FJ205875 | USA | 1995 |
| 1V | AF311956 | -N/A- | 1997 |
| 1V | EU482567 | USA | 1998 |
| 1V | AB519681 | Brazil | 2001 |
| 1V | DQ285559 | Reunion | 2004 |
| 1V | HQ166037 | Mexico | 2008 |
| 1V | KJ189351 | Puerto Rico | 2012 |
| 1V | AY277666 | -N/A- | -N/A- |
| 1V | AY206457 | -N/A- | -N/A- |
| 1V | AY277665 | -N/A- | -N/A- |

| Dengue Virus 2 Genotype | Accession Number | Country | Year |
| --- | --- | --- | --- |
| 2I (American) | EU056812 | Puerto Rico | 1977 |
| 2I (American) | EU056811 | Peru | 1995 |
| 2I (American) | AF100469 | -N/A- | -N/A- |
| 2II (Cosmopolitan) | EU081180 | Singapore | 2005 |
| 2II (Cosmopolitan) | EU081179 | Singapore | 2005 |
| 2II (Cosmopolitan) | EU081177 | Singapore | 2005 |
| 2II (Cosmopolitan) | EU179859 | Brunei | 2006 |
| 2II (Cosmopolitan) | KC762660 | Indonesia | 2007 |
| 2II (Cosmopolitan) | KC762669 | Indonesia | 2007 |
| 2II (Cosmopolitan) | KC762680 | Indonesia | 2010 |
| 2II (Cosmopolitan) | KM279597 | Singapore | 2012 |
| 2III (SE Asian-America) | EU482582 | USA | 1989 |
| 2III (SE Asian-America) | GQ868540 | Venezuela | 1990 |
| 2III (SE Asian-America) | GQ398290 | Puerto Rico | 1994 |
| 2III (SE Asian-America) | AY702036 | Cuba | 1997 |
| 2III (SE Asian-America) | AB122020 | Dominican Republic | 2001 |
| 2III (SE Asian-America) | FJ898461 | Belize | 2002 |
| 2III (SE Asian-America) | EU687216 | USA | 2005 |
| 2III (SE Asian-America) | EU687217 | USA | 2005 |
| 2III (SE Asian-America) | GQ199868 | Nicaragua | 2007 |
| 2III (SE Asian-America) | HQ999999 | Guatemala | 2009 |
| 2III (SE Asian-America) | AF489932 | -N/A- | -N/A- |
| 2III (SE Asian-America) | M20558 | -N/A- | -N/A- |
| 2IV (Asian II) | KF744406 | Philippines | 1995 |
| 2IV (Asian II) | KF744407 | Philippines | 1996 |
| 2IV (Asian II) | HQ891023 | Taiwan | 2008 |
| 2IV (Asian II) | AF204177 | China | -N/A- |
| 2IV (Asian II) | AF038403 | -N/A- | -N/A- |
| 2V (Asian I) | DQ181806 | Thailand | 1974 |
| 2V (Asian I) | DQ181805 | Thailand | 1979 |
| 2V (Asian I) | DQ181804 | Thailand | 1984 |
| 2V (Asian I) | DQ181802 | Thailand | 1988 |
| 2V (Asian I) | GQ868545 | -N/A- | 1996 |
| 2V (Asian I) | DQ181798 | Thailand | 1999 |
| 2V (Asian I) | DQ181797 | Thailand | 2001 |
| 2V (Asian I) | FM210211 | Viet Nam | 2003 |
| 2V (Asian I) | GU131896 | Cambodia | 2007 |
| 2VI (Sylvatic) | EF105387 | Nigeria | 1966 |
| 2VI (Sylvatic) | EF105379 | Malaysia | 1970 |
| 2VI (Sylvatic) | EF105382 | Burkina Faso | 1980 |
| 2VI (Sylvatic) | EF105389 | Senegal | 1999 |
| 2VI (Sylvatic) | FJ467493 | Malaysia | 2008 |

| Dengue Virus 3 Genotype | Accession Number | Country | Year |
| --- | --- | --- | --- |
| 3I | AY858039 | Indonesia | 1998 |
| 3I | KC762682 | Indonesia | 2007 |
| 3I | KC762681 | Indonesia | 2007 |
| 3I | KC762686 | Indonesia | 2007 |
| 3I | KC762684 | Indonesia | 2007 |
| 3I | KC762687 | Indonesia | 2008 |
| 3I | KC762689 | Indonesia | 2008 |
| 3I | KC762688 | Indonesia | 2008 |
| 3I | KC762690 | Indonesia | 2008 |
| 3I | KC762685 | Indonesia | 2008 |
| 3I | KC762691 | Indonesia | 2008 |
| 3I | AY858037 | Indonesia | -N/A- |
| 3II | AY876494 | Thailand | 1994 |
| 3II | AY923865 | Thailand | 1994 |
| 3II | AY766104 | Singapore | 1995 |
| 3II | DQ675528 | Taiwan | 1998 |
| 3II | DQ675525 | Taiwan | 1998 |
| 3II | DQ675532 | Taiwan | 1998 |
| 3II | AY496871 | Bangladesh | 2002 |
| 3II | AY496877 | Bangladesh | 2002 |
| 3II | AY496874 | Bangladesh | 2002 |
| 3II | AY496873 | Bangladesh | 2002 |
| 3III | JQ411814 | Sri Lanka | 1989 |
| 3III | AY099337 | Martinique | 1999 |
| 3III | FJ639747 | Venezuela | 2000 |
| 3III | FJ547071 | USA | 2000 |
| 3III | FJ898458 | Peru | 2002 |
| 3III | AY679147 | Brazil | 2002 |
| 3III | EU529702 | USA | 2003 |
| 3III | FJ898442 | Mexico | 2007 |
| 3III | GQ868578 | Colombia | 2007 |
| 3III | AY099336 | Sri Lanka | -N/A- |
| 3III | AY770511 | India | -N/A- |
| 3V | EF629370 | Brazil | 2002 |
| 3V | AF317645 | China | -N/A- |

| Dengue Virus 4 Genotype | Accession Number | Country | Year |
| --- | --- | --- | --- |
| 4I | GQ868594 | Philippines | 1956 |
| 4I | AY618991 | Thailand | 1977 |
| 4I | FJ196850 | China | 1990 |
| 4I | AY618992 | Thailand | 2001 |
| 4I | KF041260 | Pakistan | 2009 |
| 4I | JQ513345 | Brazil | 2011 |
| 4II | GU289913 | Colombia | 1982 |
| 4II | JF262782 | Haiti | 1994 |
| 4II | AY762085 | -N/A- | 1995 |
| 4II | JF262781 | Venezuela | 1995 |
| 4II | GQ252675 | USA | 1995 |
| 4II | FJ024476 | Colombia | 1997 |
| 4II | FJ882581 | Venezuela | 2007 |
| 4II | JN983813 | Brazil | 2010 |
| 4II | AF326573 | -N/A- | -N/A- |
| 4III | AY618988 | Thailand | 1997 |
| 4III | AY618989 | Thailand | 1997 |
| 4IV | JF262780 | Malaysia | 1973 |
| 4IV | EF457906 | Malaysia | 1975 |
