# Supplementary material for: A computational method for the identification of Dengue, Zika and Chikungunya virus species and genotypes
Source: PLoS Negl Trop Dis. 2019 May 8;13(5):e0007231. doi: 10.1371/journal.pntd.0007231 (PMC6527240; doi:10.1371/journal.pntd.0007231)
Supplement: S2 Table — Phylogenetic signal was calculated separately per protein by the likelihood mapping method implemented in the software TreePuzzle. Likelihood mapping analysis computes the likelihood of the three possible trees that can be constructed from all possible inter-genotype quartets of taxa. The results for the resolved quartets and unresolved quartets are shown in the table, while the partially resolved quartets are not listed (can be obtained by 100%—(un)resolved quartets). Partially resolved quartets represent the quartets for which conflicting phylogenetic signal or potential recombination is present. Genomic regions for which the percentage of resolved quartets is higher than 90% are shaded in orange and are considered to be characterized by sufficient phylogenetic signal. (DOCX) [file pntd.0007231.s004.docx]

**S2 Table:** Phylogenetic signal for DENV (DENV-1 to DENV-4), ZIKV and CHIKV sub-genomic regions. Phylogenetic signal was calcualted by a likelihood-mapping method implemented in the TreePuzzle software. Likelihood mapping analysis calculates the likelihood of the three possible trees that can be constructed from all possible quartets of taxa. The results for the resolved quartets and unresolved quartets are shown in the table. Genomic regions, which the percentage of resolved quartets is > 90% are shaded in orange.

|  | **DENV-1** | | **DENV-2** | | **DENV-3** | | **DENV-4** | |
| --- | --- | --- | --- | --- | --- | --- | --- | --- |
|  | Resolved | Unresolved | Resolved | Unresolved | Resolved | Unresolved | Resolved | Unresolved |
| **C** | 54.5% | 42,3% | 68,3% | 25,8% | 86,1% | 9,8% | 78,3% | 15,5% |
| **E** | 93.2% | 3,3% | 95,3% | 2,5% | 97,2% | 1,3% | 92,9% | 3,4% |
| **M** | 75.3% | 20,2% | 87,6% | 8,8% | 88,2% | 7,3% | 83,4% | 12,2% |
| **NS1** | 92.2% | 4,5% | 89,4% | 8,1% | 96,7% | 1,3% | 95,4% | 2,3% |
| **NS2A** | 82.5% | 15,5% | 87,7% | 9,4% | 88,5% | 8,3% | 87,3% | 8,5% |
| **NS2B** | 79.0% | 19,2% | 83,0% | 14,4% | 89,2% | 6,3% | 82,5% | 12,7% |
| **NS3** | 94,6% | 2,8% | 94,5% | 2,6% | 97,2% | 1,3% | 95,0% | 2,7% |
| **NS4A** | 73,8% | 19,9% | 78,1% | 19,8% | 88,7% | 8,4% | 84,7% | 9,9% |
| **NS4B** | 85,2% | 12,7% | 90,2% | 5,7% | 92,2% | 4,1% | 83,5% | 10,6% |
| **NS5** | 94,7% | 2,0% | 94,0% | 3,2% | 98,1% | 0,7% | 97,0% | 1,4% |
|  |  |  |  |  |  |  |  |  |
|  | **ZIKV** | |  |  | **CHIKV** | |  |  |
|  | Resolved | Unresolved |  |  | Resolved | Unresolved |  |  |
| **C** | 78.2% | 3.9% |  | **NSP1** | 52.2% | 47.7% |  |  |
| **E** | 94.3% | 1.5% |  | **NSP2** | 52.1% | 47.9% |  |  |
| **M** | 81.6% | 4.8% |  | **NSP3** | 62.3% | 19.6% |  |  |
| **NS1** | 91.9% | 1.8% |  | **NSP4** | 52.7% | 46.8% |  |  |
| **NS2A** | 93.5% | 2.3% |  | **CAP** | 56.9% | 35.6% |  |  |
| **NS2B** | 85.4% | 1.1% |  | **E3** | 58.2% | 26.9% |  |  |
| **NS3** | 94.0% | 1.7% |  | **E2*** | 77.1% | 13.1% |  |  |
| **NS4A** | 90.8% | 3.7% |  | **E1** | 53.1% | 41% |  |  |
| **NS4B** | 93.2% | 2.0% |  |  |  |  |  |  |
| **NS5** | 96.3% | 2.4% |  |  |  |  |  |  |
