# Supplementary material for: A computational method for the identification of Dengue, Zika and Chikungunya virus species and genotypes
Source: PLoS Negl Trop Dis. 2019 May 8;13(5):e0007231. doi: 10.1371/journal.pntd.0007231 (PMC6527240; doi:10.1371/journal.pntd.0007231)
Supplement: S3 Table — The new classification method consists of 2 parts: determining the species (and for DENV also the serotype) using a BLAST procedure, followed by determining the genotype using an automated phylogenetic method. Our method was able to assign all sequences in the whole-genome validation dataset to the right species and DENV serotype. Therefore, in this table, we focus on the classification performance with respect to genotype assignment, based on the output of the BLAST step (i.e. a dataset of the proper species and serotype). The classification results were compared to manual phylogenetic analysis. Column names: TP = total positives, TN = total negatives, FP = false positive, FN = false negative, SENS = sensitivity, SPEC = specificity, ACC = accuracy. (DOCX) [file pntd.0007231.s005.docx]

**S3 Table:** Evaluation of the automated phylogenetic method to classify Dengue, Chikungunya and Zika viruses complete genomes. The classification results were compared to manual phylogenetic analysis. In TP, total positives, TN, total negatives, FP, false positive, FN, False negative, Sens, sensitivity, Spec, specificity, PPV, positive predicted value, NPV, negative predict value. * Manual phylogenetic analysis could also not classify these sequences. Results from the evaluation of sequences from close related viruses, other sub-genomic regions and next generation sequences (NGS) is find in supplementary information.

| **Virus species** | **Known** | **TP** | **TN** | **FP** | **FN** | **SENS** | **SPEC** | **ACC** |
| --- | --- | --- | --- | --- | --- | --- | --- | --- |
| **Dengue Virus Serotype 1** | 1688 | 1688 | 3496 | 0 | 0 | 100,0% | 100,0% | 100,0% |
| 1i | 1151 | 1151 | 4033 | 0 | 0 | 100,0% | 100,0% | 100,0% |
| 1ii | 28 | 28 | 5156 | 0 | 0 | 100,0% | 100,0% | 100,0% |
| 1iii | 26 | 26 | 5158 | 0 | 0 | 100,0% | 100,0% | 100,0% |
| 1iv | 63 | 63 | 5121 | 0 | 0 | 100,0% | 100,0% | 100,0% |
| 1v | 413 | 413 | 4771 | 0 | 0 | 100,0% | 100,0% | 100,0% |
| Genotype could not be assigned* | 7 |  |  |  |  |  |  |  |
| **Dengue Virus Serotype 2** | 1317 | 1317 | 3867 | 0 | 0 | 100,0% | 100,0% | 100,0% |
| 2i (American) | 47 | 47 | 5137 | 0 | 0 | 100,0% | 100,0% | 100,0% |
| 2ii (Cosmopolitan) | 245 | 245 | 4939 | 0 | 0 | 100,0% | 100,0% | 100,0% |
| 2iii (SE Asian-America) | 649 | 649 | 4535 | 0 | 0 | 100,0% | 100,0% | 100,0% |
| 2iv (Asian II) | 41 | 41 | 5143 | 0 | 0 | 100,0% | 100,0% | 100,0% |
| 2v (Asian I) | 317 | 317 | 4867 | 0 | 0 | 100,0% | 100,0% | 100,0% |
| 2vi (Sylvatic) | 17 | 17 | 5167 | 0 | 0 | 100,0% | 100,0% | 100,0% |
| Genotype could not be assigned* | 1 |  |  |  |  |  |  |  |
| **Dengue Virus Serotype 3** | 897 | 897 | 4287 | 0 | 0 | 100,0% | 100,0% | 100,0% |
| 3i | 68 | 68 | 5116 | 0 | 0 | 100,0% | 100,0% | 100,0% |
| 3ii | 190 | 190 | 4994 | 0 | 0 | 100,0% | 100,0% | 100,0% |
| 3iii | 620 | 620 | 4564 | 0 | 0 | 100,0% | 100,0% | 100,0% |
| 3v | 18 | 18 | 5166 | 0 | 0 | 100,0% | 100,0% | 100,0% |
| Genotype could not be assigned* | 1 |  |  |  |  |  |  |  |
| **Dengue Virus Serotype 4** | 216 | 216 | 4968 | 0 | 0 | 100,0% | 100,0% | 100,0% |
| 4i | 23 | 23 | 5161 | 0 | 0 | 100,0% | 100,0% | 100,0% |
| 4ii | 187 | 187 | 4997 | 0 | 0 | 100,0% | 100,0% | 100,0% |
| 4iii | 2 | 2 | 5182 | 0 | 0 | 100,0% | 100,0% | 100,0% |
| 4iv | 3 | 3 | 5181 | 0 | 0 | 100,0% | 100,0% | 100,0% |
| Genotype could not be assigned* | 1 |  |  |  |  |  |  |  |
| Total | 4118 |  |  |  |  |  |  |  |
| **Chikungunya Virus** |  |  |  |  |  |  |  |  |
| East-Central-South-African | 274 | 274 | 4910 | 0 | 0 | 100,0% | 100,0% | 100,0% |
| Asian and Caribbean | 364 | 364 | 4820 | 0 | 0 | 100,0% | 100,0% | 100,0% |
| West African | 15 | 15 | 5169 | 0 | 0 | 100,0% | 100,0% | 100,0% |
| Total | 653 |  |  |  |  |  |  |  |
| **Zika Virus** |  |  |  |  |  |  |  |  |
| African | 26 | 26 | 5158 | 0 | 0 | 100,0% | 100,0% | 100,0% |
| Asian | 387 | 387 | 4797 | 0 | 0 | 100,0% | 100,0% | 100,0% |
| Total | 413 |  |  |  |  |  |  |  |
| **Total sequences** | **5184** |  |  |  |  |  |  |  |
